# Contextual factors associated with health care service utilization for children with acute childhood illnesses in Nigeria

**DOI:** 10.1371/journal.pone.0173578

**Published:** 2017-03-15

**Authors:** Sulaimon T. Adedokun, Victor T. Adekanmbi, Olalekan A. Uthman, Richard J. Lilford

**Affiliations:** 1 Warwick-Centre for Applied Health Research and Delivery (WCAHRD), Division of Health Sciences, University of Warwick Medical School, Coventry, United Kingdom; 2 Department of Demography and Social Statistics, Obafemi Awolowo University, Ile Ife, Nigeria; 3 NIHR Collaboration for Leadership in Applied Health Research and Care, West Midlands (CLAHRC WM), University of Warwick Medical School, Coventry, United Kingdom; Centre Hospitalier Universitaire Vaudois, FRANCE

## Abstract

**Objective:**

To examine the independent contribution of individual, community and state-level factors to health care service utilization for children with acute childhood illnesses in Nigeria.

**Materials and methods:**

The study was based on secondary analyses of cross-sectional population-based data from the 2013 Nigeria Demographic and Health Survey (DHS). Multilevel logistic regression models were applied to the data on 6,427 under-five children who used or did not use health care service when they were sick (level 1), nested within 896 communities (level 2) from 37 states (level 3).

**Results:**

About one-quarter of the mothers were between 15 and 24 years old and almost half of them did not have formal education (47%). While only 30% of the children utilized health service when they were sick, close to 67% lived in the rural area. In the fully adjusted model, mothers with higher education attainment (Adjusted odds ratio [aOR] = 1.63; 95% credible interval [CrI] = 1.31–2.03), from rich households (aOR = 1.76; 95% CrI = 1.35–2.25), with access to media (radio, television or magazine) (aOR = 1.18; 95% CrI = 1.08–1.29), and engaging in employment (aOR = 1.18; 95% CrI = 1.02–1.37) were significantly more likely to have used healthcare services for acute childhood illnesses. On the other hand, women who experienced difficulty getting to health facilities (aOR = 0.87; 95% CrI = 0.75–0.99) were less likely to have used health service for their children.

**Conclusions:**

Our findings highlight that utilization of healthcare service for acute childhood illnesses was influenced by not only maternal factors but also community-level factors, suggesting that public health strategies should recognise this complex web of individual composition and contextual composition factors to guide provision of healthcare services. Such interventions could include: increase in female school enrolment, provision of interest-free loans for small and medium scale enterprises, introduction of mobile clinics and establishment of more primary health care centres.

## Introduction

Although under-five mortality has reduced globally, the situation in sub-Saharan Africa is still a major concern. Sub-Saharan Africa makes up less than a sixth of the world population yet about half of all under-five deaths took place in this region in 2015 [1]. The leading causes of mortality in young children are infectious diseases such as diarrhoea and malaria. Ninety percent of the world’s under-five deaths due to diarrhoea and even higher proportion of the deaths due to malaria occurred in sub-Saharan Africa [2]. Nigeria mirrors the regional picture; 11% of deaths among under-five were caused by diarrhoea and 20% were linked to malaria [3, 4].

Nigerian governments have responded to the challenge and embarked on policies to promote child health and make health care services available and affordable. Such policies include National Acute Respiratory Infections Programme of 1991, Maternal and Child Health Policy of 1994, National Immunization Policy and Standards Practice of 1996 and Breastfeeding Policy of 1999 [5]. In addition, government ratified and adopted the International Child rights policy which highlight, among other things, the provision of medical and health care for all children and tackling diseases and malnutrition [5]. By putting these initiatives in place, the expectation was that mothers and caregivers would have the opportunity to utilize health care services for themselves and their children. However, evidence has shown that such services are poorly accessed. Instead, mothers or caregivers more often than not resort to traditional healers, over-the-counter drug peddlers or self-medication [6–8]. This poor patronage of health care services constitutes a great impediment to child health improvement particularly in respect of the proposed Sustainable Development Goal (SDG). The SDG aims at ending preventable deaths of new-borns and under-five by 2030. To achieve this, all countries are planning to reduce under-five mortality from what it is now to as low as 25 deaths per 1000 live births [1].

Meanwhile, attempts have been made through different studies to identify the factors that are responsible for the poor utilization of health care services for children [9–16]. These studies have focused mainly on individual factors such as age, education, marital status, occupation and income, ignoring factors operating at the community and broader levels. The aim of this study was to examine the association between individual, community and state level factors and health care utilization for Nigerian children with frailer conditions; diarrhoea and fever or cough.

## Materials and methods

### Setting

Nigeria is located in the western part of Africa and shares borders with Niger, Chad, Cameroon and Benin. It comprises 36 states, 6 geo-political zones and its population at the last Census exercise was over 140 million [17]. At independence in 1960, the main income for the country was generated from agriculture. However, attention has shifted from agriculture since the discovery of oil. The earnings from the sale of oil contributed immensely to developments in the country. Over the years, the health sector witnessed major developments in terms of establishment of hospitals, provision of equipment and drugs and introduction of programmes to promote the well-being of the citizens. Presently, the country operates a National Health Insurance Scheme which aims at making people have access to health services, relieving family of high medical expenses and ensuring equitable distribution of health care services [18].

The scheme covers both the formal and informal sectors. The formal sector comprises the public sector, organized private sector and the armed forces including the police and uniformed services. In the public sector scheme, 3.25% and 1.75% of the employee’s salary are paid by the employer and employee respectively [18]. In the private sector category, the employee pays 5% while the employer pays 10%. These contributions are meant to cover the health care expenses incurred by the employee, his/her spouse and four children below 18 years of age. The scheme has three categories in the informal sector: the tertiary social health insurance programme which makes provision to cater for the health care expenses of students in higher institutions; community-based social health insurance programme which is a voluntary scheme that enables communities to enjoy health services through the payment of flat rate per household or individual household member and; public primary school social health insurance which is targeted at primary school pupils from middle and lower socioeconomic status.

### Study design

This study was based on analyses of secondary data set from the Nigeria Demographic and Health Survey (DHS) 2013 which is cross-sectional and covers all the geo-political zones in the country.

### Sampling technique

Details of the methods used in the DHS have been published elsewhere [17]. Briefly, the survey involved a three-stage cluster sampling technique. Nigeria was divided into 36 States and the Federal Capital Territory (FCT), Abuja making 37 districts in total. The primary sample unit (PSU) was based on 2006 Nigeria population census enumeration areas (EAs). The first stage involved selecting 896 localities (clusters). In the second stage, one EA was randomly selected from most localities. A total of 904 EAs were selected, with 372 in urban areas and 532 in rural areas. The third stage involved the selection of a fixed number of 45 households in every urban and rural geographical area. The total number of selected households was 40,680, with urban areas accounting for 16,740 and 23,940 from rural areas.

### Data collection

The methods for data collection have been published elsewhere [17]. In brief, data were collected through household visitation and interviews with individual participants in the selected localities. Information on socio-demographic characteristics, wealth, reproduction, child health, knowledge of HIV/AIDS, domestic violence, household and environmental characteristics was obtained from the participants.

### Ethical consideration

This study was based on secondary analysis of existing survey datasets from the archive of the DHS Program who granted us permission for its usage after all the identifying information have been removed. The instruments and conduct of the 2013 Nigeria DHS was approved by the Institutional Review Board (IRB) of ICF Macro International in the United States and Nigeria Health Research Ethics Committee (NHREC) of the Federal Ministry of Health (FMOH). This research is limited to the use of previously collected anonymised data.

### Outcome variable

Users/non users of health services; children under-five who had episode of diarrhea and/or fever or cough in the preceding 2 weeks before the survey and who sought consultation from a health care provider (either public or private) were defined as ‘users’; not seeking care were categorized as ‘non-users’.

### Independent variables

**Fig 1:**
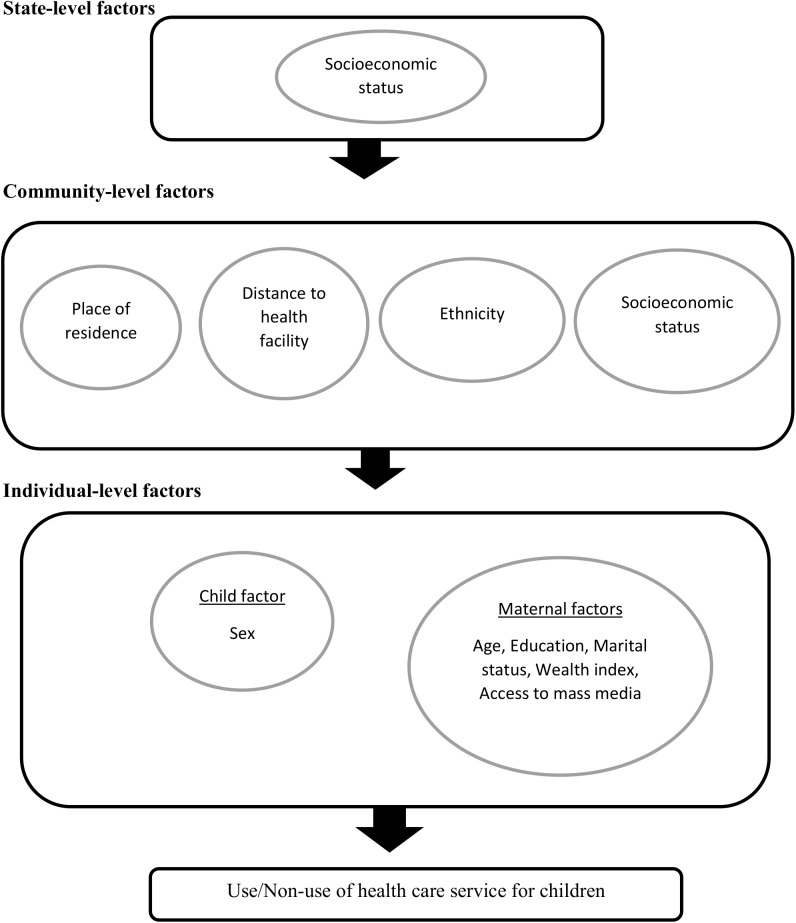
Conceptual framework showing the factors influencing health care service utilization for children. Figure adapted from Andersen RM. Revisiting the Behavioral Model and Access to Medical Care: Does it Matter? Journal of Health and Social Behavior. 1995; 36: 1–10

We adopted behavioural model developed by Andersen to understand the dynamic inter-relations among people and environmental factors associated with health services use for a sick child (**Fig 1**) [19]. We subsequently grouped such factors into individual-, community- and state-level factors.

### Individual-level factors

In this study, we considered the following variables: age of mother, educational attainment and marital status, mother’s occupation, sex of the child, wealth status and media access. Age of mother (the respondent) was categorised as 15–24, 25–34 and 35–49. The level of education attained by mother was defined as no education, primary, and secondary or higher education. Respondents' current occupation was categorised into unemployed and employed (professional, technical and managerial, services, agricultural, skilled and unskilled manual and others). Marital status was dichotomized as ever married (i.e. currently married, living with partner, widowed, divorced, separated) and never married. Sex of the child was categorised into Male/Female. Wealth index is measured in the DHS surveys in terms of assets, rather than income. Ownership of consumer items such as a radio or car as well as dwelling characteristics such as floor or roof type, place of cooking, cooking fuel, electricity, toilet facilities and water source were the items that constituted the concept of poverty. This concept has been used by the World Bank to categorise households and their members into different wealth quintiles, through the use of principal components analysis (PCA) [20, 21]. For easy analysis, we re-categorized the weighted scores of five quintiles to three tertiles to allow for nonlinear effects and provide results that would be more readily interpretable in the policy domain. The resultant three tertiles expressed as categorical variables include poor, middle and rich. Access to media was measured as a set of additive scale (from 0 to 3) that counted the number of domains in which each of the respondents was regarding having access to various types of media (radio, television and magazine). This resulted into the following categories: no access, have access to 1 outlet, have access to 2 outlets and have access to all outlets.

### Community-level factors

At community level, we included place of residence, distance to health facility, community socioeconomic status and ethnicity diversity index. Place of residence was categorized into urban and rural. Distance to health facility was grouped into two: those who experienced difficulty in reaching health facility were categorised as ‘a problem’ and those who did not experience difficulty were categorised as ‘not a problem’. Community socioeconomic disadvantage was an index created from the compositional education, wealth and occupation of people within the same PSU. Community socioeconomic disadvantage was obtained through a principal component that consisted of the proportion of respondents with: no education (illiterate), unemployed, and living below the poverty level (asset index below 20% poorest quintile) in the same PSU. This resulted in the generation of a standardized score with mean 0 and standard deviation 1; with higher scores indicative of lower socioeconomic position. We divided the resultant scores into three equal tertiles.

The ethnicity of the children was computed by using ethnicity diversity index. We obtained the index by using a formula derived by Vyas and Kumaranayake [22] that captures both the number of different ethnic groups in an area and the relative representation of each group as follows

$$\text{Ethnic diversity index}=1-\sum_{i=1}^{n}{\left[\frac{x_{i}}{y}\right]}^{2}$$ (1)

Where: *x*_*i*_ = population of ethnic group i of the area, y = total population of the area, n = number of ethnic groups in the area.

Scores can range from 0 to approximately 1. For easy interpretation, we multiplied each diversity index by 100; the higher the index score, the greater the diversity in the area. An area with zero diversity indicates that all the people in the area belong to one ethnic group. As the index moves close to 100, the population becomes more evenly distributed into ethnic groups.

### State-level factors

State socioeconomic disadvantage was an index created from the compositional education, wealth and occupation. State socioeconomic disadvantage was operationalized with a principal component comprised of the proportion of respondents with: no education (illiterate), unemployed, and living below the poverty level (asset index below 20% poorest quintile) within the state. A standardized score with mean 0 and standard deviation 1 was generated from this index; with higher scores indicative of lower socioeconomic position. We divided the resultant scores into three tertiles; tertile 1(least economically disadvantaged), tertile 2 and tertile 3(most economically disadvantaged)

### Statistical analyses

#### Descriptive analyses

In the descriptive statistics, the respondents’ characteristics at different levels were expressed as numbers and percentages.

#### Modelling approaches

We specified a three-level model with individuals clustered within the communities and the communities clustered within the states in order to estimate the effects of variables at all three levels on utilization of health services. We constructed four models. The first model is a univariable model of the individual-level factors. The second model contained community-level variables while the third model contained the state-level variable at the univariable level. The fourth model adjusted for the individual-level, community-level and state-level variables respectively. P value of < 0.05 was used to define statistical significance.

#### Fixed effects (measures of association)

We presented the results of fixed effects as odds ratios (OR) with their corresponding 95% credible intervals (CrI).

#### Random effects (measures of variation)

Random effects were measured through intra-cluster correlation (ICC), variance partition coefficient (VPC) and median odds ratio (MOR). Median odds ratio, which reflects the unexplained cluster heterogeneity, measures the area variance as odds ratios. Details of the procedure used for calculating MOR have been published elsewhere [23, 24].

#### Model fit and specifications

While we applied Bayesian Deviance Information Criterion (DIC) to assess the goodness-of-fit of the model, Variance Inflation Factor (VIF) was used to check for multicollinearity among the independent variables. All multilevel modelling operations were performed using MLwiN 2.36 [25] calling Stata statistical software for windows version 14 [26]. The Bayesian approach with Markov Chain Monte Carlo (MCMC) estimation was used [27] for the multilevel logistic regression models.

## Results

### Sample characteristics

Table 1 presents the descriptive statistics for the final sample included in this study. For this analysis, we analysed information on 6,427 children under-five years of age (Level 1), nested within 896 communities (Level 2), from 37 states (Level 3) in Nigeria. As shown in Table 1, about 70% of the children did not utilize health service while only 30% utilized health service when they were sick. Distribution of children in terms of their gender was fairly evenly distributed. About one-quarter of the mothers were aged between 15 and 24 years (26%), almost half had no formal education (45%), and one-third were not employed (33%). The preponderance of the mothers were ever married (98%). Slightly more than one-third (36%) of the mothers had no access to any form of media (radio, television and magazines). Most of the children were living in rural areas (67%). One-third of the women experienced difficulty getting to the health facility.

**Table 1:** Percentage distribution of participants’ characteristics at different levels.

Variable	Number (%)
**Seek treatment for sick child**	
No	4,491 (69.9)
Yes	1,936 (30.1)
**Individual level factors**	**(n = 6,427)**
Sex	
Male	3,288 (51.2)
Female	3,139 (48.8)
Maternal age (years)	
15–24	1,690 (26.3)
25–34	3,179 (49.5)
35–49	1,558 (24.2)
Maternal education	
No education	2,919 (45.4)
Primary	1,360 (21.2)
Secondary or higher	2,148 (33.4)
Maternal occupation	
Not working	2,107 (32.8)
Working	4,320 (67.2)
Marital status	
Never married	150 (2.3)
Ever married	6,277 (97.7)
Wealth index of family	
Poorer	2,143 (33.3)
Middle	2,143 (33.3)
Richer	2,141 (33.3)
Access to Media	
No access	2,340 (36.4)
Have access to 1 outlet	1,581 (24.6)
Have access to 2 outlets	1,765 (27.5)
Have access to all outlets	741 (11.5)
**Community-level factors**	**(n = 896)**
Place of residence	
Rural	4,315 (67.1)
Urban	2,112 (32.9)
Distance to health facility	
Not a problem	4,331(67.4)
A problem	2,096(32.6)
Ethnicity diversity index, mean (SD)	2.6 (2.9)
Socioeconomic disadvantage	
Tertile 1 (least disadvantaged)	2,143 (33.3)
Tertile 2	2,142 (33.3)
Tertile 3 (most disadvantaged)	2,142 (33.3)
**State-level factors**	**(n = 37)**
Socioeconomic disadvantage	
Tertile 1 (least disadvantaged)	2,199 (34.2)
Tertile 2	2,162 (33.6)
Tertile 3 (most disadvantaged)	2,066 (32.2)

### Measures of associations (fixed effects)

The results of different models are shown in Table 2, including the fully adjusted model controlling for the effects of individual-, community- and state-level factors (Model 4). The odds of using health services for sick children increased with maternal level of education, such that women with secondary or higher education were 1.63 times more likely to have used health services for their sick children compared with those mothers with no education (OR = 1.63, 95% CrI 1.31 to 2.03). Women who are working were 1.18 times more likely to have used health service for their children compared to non-working women (OR = 1.18, 95% CrI 1.02 to 1.37). Women from rich households were 1.76 times more likely to have used health services for their sick children compared to those from poor households (OR = 1.76, 95% CrI 1.35 to 2.25). Those who had access to media were 1.18 times more likely to have used health service for their children compared to those who had no media access (OR = 1.18, 95% CrI 1.08 to 1.29). Women who experienced problems getting to the health facility were less likely to have used health service for their children compared to women who did not experience any problem (OR = 0.87, 95% CrI 0.75 to 0.99). Contrary to expectation, health service was sought for children living in the more socioeconomically disadvantaged communities and states compared to children living in the least socioeconomically disadvantaged communities and states.

**Table 2:** Factors associated with health care utilisation identified by multilevel multivariate logistic regression models.

Variable	Model 1^a^	Model 2^b^	Model 3^c^	Model 4^d^
FIXED-EFFECTS	OR (CrI)	OR (CrI)	OR (CrI)	aOR (CrI)
**Individual level factors**				
Male (vs female)	1.07(0.95–1.20)			1.07(0.95–1.19)
Age of mothers in years				
15–24	1 (reference)			1(reference)
25–34	1.11(0.95–1.29)			1.12(0.96–1.32)
35–49	1.15(0.95–1.36)			1.16(0.95–1.40)
Education attainment of mother				
No education	1 (reference)			1 (reference)
Primary	1.15(0.94–1.39)			1.18(0.97–1.43)
Secondary or higher	1.59(1.28–1.98)			1.63(1.31–2.03)
Occupation of mother				
Not working	1 (reference)			1(reference)
Working	1.14(1.01–1.31)			1.18(1.02–1.37)
Marital status				
Never married	1 (reference)			1(reference)
Ever married	1.31(0.78–1.73)			1.28(0.78–2.04)
Wealth index of family				
Poor	1 (reference)			1 (reference)
Middle	1.48(1.21–1.77)			1.43(1.18–1.75)
Rich	1.85(1.44–2.35)			1.76(1.35–2.25)
Access to Media				
No	1 (reference)			1 (reference)
Yes	1.19(1.10–1.28)			1.18(1.08–1.29)
**Community-level factors**				
Rural (vs. urban)		1.52(1.21–1.85)		1.14(0.88–1.45)
Distance to health facility				
Not a problem		1 (reference)		1 (reference)
A problem		0.81(0.70–0.95)		0.87 (0.75–0.99)
Ethinicity diversity index		1.01(0.98–1.05)		1.01(0.97–1.04)
Socioeconomic disadvantage				
Tertile 1 (least disadvantaged)		1 (reference)		1(reference)
Tertile 2		1.26(0.99–1.59)		1.48(1.17–1.85)
Tertile 3 (most disadvantaged)		0.73(0.53–1.007)		1.24(0.82–1.73)
**State-level factors**				
Socioeconomic disadvantage				
Tertile 1 (least disadvantaged)			1 (reference)	1(reference)
Tertile 2			1.12(0.65–1.61)	1.25(0.76–1.88)
Tertile 3 (most disadvantaged)			1.11(0.59–2.01)	2.01(1.04–3.17)

^a^Model 1 is univariable model for sex of child, age, education, occupation, marital status, wealth status of family and access to media.

^b^Model 2 is univariable model for residency, distance to health facility, ethnicity diversity index, and community socioeconomic factors.

^c^Model 3 is univariable model for state-level socioeconomic factors.

^d^Model 4 is multivariable model for model 1, Model 2 and Model 3 respectively.

aOR; Adjusted odds ratio, CrI; credible interval.

### Measures of variations (random effects)

As shown in Table 3, in Model 1 (unconditional model), there was a significant variation in the odds of reporting health services utilization for sick children across the states (*σ*^2^ = 0.260, 95% CrI 0.123 to 0.468) and across the communities (*σ*^2^ = 0.698, 95% CrI 0.537 to 0.897). The intra-state and intra-community correlation coefficients are 6.1 and 22.6 respectively. From the full model (Model 5), the median odds ratios (MOR) for the communities and states are 2.02 and 1.65 respectively.

**Table 3:** Results from random intercept model–measures of variation.

	Model 1^a^	Model 2^b^	Model 3^c^	Model 4^d^	Model 5^e^
Measures of variation					
State level					
Variance (SE)	0.260(0.123–0.468)	0.369(0.195–0.642)	0.279(0.141–0.504)	0.292(0.148–0.527)	0.280(0.138–0.522)
Explained variation (%)	Reference	-41.9	-7.4	-11.9	-7.5
Intra-state correlation (%)	6.13	8.70	6.79	6.81	6.80
MOR	1.62	1.78	1.65	1.67	1.65
Community level					
Variance (SE)	0.698(0.537–0.897)	0.587(0.427–0.792)	0.551(0.404–0.716)	0.697(0.546–0.869)	0.551(0.391–0.719)
Explained variation (%)	Reference	15.9	21.0	0.1	21.1
Intra-community correlation (%)	22.56	22.53	20.17	23.12	20.16
MOR	2.21	2.07	2.02	2.21	2.02
Model fit statistics					
Bayesian DIC	7268	7166	7249	7269	7170

^a^Model 1 is empty model, a baseline with no independent variable.

^b^Model 2 is univariable model for sex of child, age, education, occupation, marital status, wealth status of family and access to media.

^c^Model 3 is univariable model for residency, distance to health facility, ethnicity diversity index, and community socioeconomic factors.

^d^Model 4 is univariable model for state-level socioeconomic factors.

^e^Model 5 is multivariable model for Model 1, Model 2, Model 3 and Model 4 respectively.

Abbreviations: SE; standard error, MOR; median odds ratio, DIC; deviation information criterion.

## Discussion

### Main findings

Using an explicit multilevel analytic framework, the study has shown that both individual composition and community contextual characteristics are important predictors of mothers’ health services utilization for sick children in Nigeria.

Our study showed that mother’s level of education is a significant predictor of health care utilization for children. This is in line with the results of previous studies [28–31]. The probability of seeking medical treatment increases with the increasing level of education. This may be attributed to the fact that educated mothers have a better understanding of seeking medical care in the event of their children being ill.

Findings from this study further underscored the role of household wealth in health care utilization. Mothers from richer households sought medical care for their children more than their counterparts from poor households. Earlier studies have established the significant influence which poverty exerts on seeking medical treatment [32–37]. Poor mothers may not have the financial capacity to convey their children to the available health facility let alone paying for the treatment. This, in most cases, results in such women embarking on home or non- medical treatment.

Results from our study also showed that working mothers utilized health service for their children more than non-working mothers. Previous studies have also supported this [38]. This may be linked to two factors: working mothers may have more financial ability to seek medical health care for their children and those who work in the formal sector, especially, may enjoy a reasonable reduction in medical expenses through the health insurance scheme.

Like most studies [39–41], we found that the more mothers are exposed to mass media, the more they utilize health care service for their children. Mass media provide readers, listeners or viewers access to information. Since information is often seen as a necessary determinant of behaviour, mothers who have access to information on good child care practices would seek medical treatment for their sick children.

Consistent with earlier findings, [32, 42–45] our study confirmed that distance to health facility is a significant determinant of health care service utilization. Women who complained of experiencing difficulty in getting to health facility are less likely to utilize health service for their children compared with other women who do not experience difficulty. Living far away from the location of health facility may force mothers to walk or travel long distance which could lead to tiredness, exposure to hazard or incurring more expenses. Such mothers would be discouraged from presenting their children for medical treatment.

The study findings, therefore, provide some significant policy implications. Health care facilities should be brought close to inhabitants of remote areas through establishment of more primary health care centres and introduction of mobile clinics. Emphasis should be placed on increase in female enrolment in schools, provision of interest-free loans for small and medium scale enterprises and the use of mass media to access information.

However, our findings are subject to some limitations. As a result of the fact that the data we have used are cross-sectional and non-experimental, causality could not be established. We were unable to take into account the impact of residential changes over time and the cumulative effects of socioeconomic environment over time. Another limitation of this study is measurement error. Measuring wealth in developing countries is challenging. Hence, we have used an indirect measure of household wealth which may be subject to criticism. It should be noted that reliable data on income and expenditure in developing countries like Nigeria is difficult to obtain. In order to overcome this challenge, we resorted to the use of an asset-based index which is generally viewed as a good proxy for measuring household wealth status. Many of the household wealth indices used for assets in DHS are more likely to be found in urban areas than in rural areas. Thus, most of the households in the rural areas will be in the lowest wealth index category even if they have other indicators of wealth (e.g., livestock or farm machinery). Despite these limitations, the strength of our study is evident. It is a large, cross-sectional and population-based study which covers all the states in the country. This allows for generalizability of the study to the entire country.

## Conclusions

This study has revealed that individual and, community- level factors are important determinants of health care service utilization for children in Nigeria. The findings emphasise the need to embark on interventions that would improve health care service utilization by mothers for their children. Such interventions should take into cognisance the individual and community characteristics. Such interventions could include, but not limited to: increase in female school enrolment, provision of interest-free loans for small and medium scale enterprises, introduction of mobile clinics and establishment of more primary health care centres. Further research efforts should focus on exploring longitudinal and experimental studies.
